# Acupuncture in Patients with Diabetic Peripheral Neuropathy-Related Complaints: A Randomized Controlled Clinical Trial

**DOI:** 10.3390/jcm12062103

**Published:** 2023-03-07

**Authors:** Joanna Dietzel, Isabel V. Habermann, Sebastian Hörder, Katrin Hahn, Gesa Meyer-Hamme, Miriam Ortiz, Kevin Hua, Barbara Stöckigt, Marie Bolster, Weronika Grabowska, Stephanie Roll, Sylvia Binting, Stefan N. Willich, Sven Schröder, Benno Brinkhaus

**Affiliations:** 1 Institute of Social Medicine, Epidemiology and Health Economics, Charité-Universitätsmedizin Berlin, Corporate Member of Freie Universität Berlin, Humboldt-Universität zu Berlin, and Berlin Institute of Health, Luisenstr. 57, 10117 Berlin, Germany; 2 Department of Neurology with Experimental Neurology, Charité-Universitätsmedizin Berlin, Corporate Member of Freie Universität Berlin, Humboldt-Universität zu Berlin, and Berlin Institute of Health, 10117 Berlin, Germany; 3 HanseMerkur Center for Traditional Chinese Medicine at the University Medical Center Hamburg-Eppendorf, Martinistrasse 64, 20251 Hamburg, Germany

**Keywords:** acupuncture, diabetic peripheral neuropathy, pain, numbness, randomized controlled trial

## Abstract

**Background**: Diabetic polyneuropathy (DPN) is a common complication of diabetes, which presents with a loss of sensorimotor function or pain. This study assessed the effectiveness and safety of acupuncture as a treatment for DPN-related complaints. **Methods**: In this randomized controlled trial, patients with type II diabetes and symptoms of neuropathy in the lower limbs were included. A total of 12 acupuncture treatments were administered over 8 weeks. The waitlist control group received the same acupuncture treatment starting at week 16 (after baseline). **Results**: A total of 62 patients were randomized (acupuncture group *n* = 31; control group *n* = 31). The primary outcome was overall complaints, and it was reduced with a significant difference of 24.7 on a VAS (CI 95% 14.8;34.7, *p* < 0.001) between both groups in favor of acupuncture. Reductions were recorded for the secondary outcomes VAS pain, neuropathic pain symptom inventory (NPSI), emotional dimensions of pain, SF-12, and diabetic peripheral neuropathic pain impact (DPNPI) after the intervention and at the follow-ups in the acupuncture group. Adverse reactions were minor and transient. **Conclusions**: Acupuncture leads to a significant and lasting reduction in DPN-related complaints when compared to routine care and is well tolerated, with minor side effects.

## 1. Introduction

According to the International Diabetes Federation, 9.3% (463 million) of the global population aged 20–79 is affected by diabetes. The numbers are increasing, and so are diabetes-related comorbidity and complications with diabetic peripheral neuropathy among them (DPN) [1]. About 13–46% of diabetic patients suffer from DPN [2].

The clinical presentation of sensorimotor neuropathy is highly variable. Patients may be asymptomatic or suffer from pain and dysesthesias in the feet and lower legs. The damage of sensory nerves may result in tingling, burning, lancinating or shooting pain, hyperalgesia, and numbness, and because of DPN, atrophy of small foot muscles can occur [3].

DPN may have distressing and incapacitating complications such as the development of foot ulceration, Charcot neuroarthropathy, the amputation of lower limbs, and a higher risk of falls and fractures [4,5]. Moreover, DPN has negative effects on mental health and psychosocial functioning, leading to depression, anxiety, and a lower health-related quality of life [5]. 

Despite the high prevalence of symptomatic DPN, there is still no satisfactory disease-modifying therapy [6]. Furthermore, according to a Cochrane meta-analysis, even enhanced glycemic control does not prevent the development and progression of DPN in type II diabetes [7].

The administration of multiple drugs, such as antidepressants, anticonvulsants, opioids, and topical treatments, is often necessary to reduce pain, albeit increasing the risk of side effects through polypharmacy. Still, adequate symptom relief is difficult to achieve. Both pain and polypharmacy have a negative impact on quality of life. Moreover, it has no effects on sensory deficits, such as numbness. Nonpharmacological approaches, such as transcutaneous electrical nerve stimulation, have been shown to produce improvements in pain outcomes [8]. Thus, further study of other possible treatments, especially regarding quality of life and the long-term effects, remains of high importance.

Acupuncture has long-lasting positive effects on chronic pain syndromes [9] and is considered a safe treatment with fewer side effects compared to many pharmaceuticals [10].

There is evidence showing a positive effect of acupuncture on neuropathies of different etiologies, including DPN-related symptoms [11,12,13]. An increase in local microcirculation through acupuncture is postulated to induce a certain degree of improved supply to the neural tissue [13]. 

The aim of the multicenter ACUpuncture in Diabetic Peripheral Neuropathy (ACUDPN) trial is to confirm the hypothesis that 12 treatments of acupuncture are safe and efficient for the treatment of DPN-related symptoms.

## 2. Materials and Methods

The trial was approved by the Ethics Committee in Berlin (EA1/183/18) and Hamburg and was executed according to the principles of Good Clinical Practice and the Declaration of Helsinki. Informed written and oral consent was given by all patients prior to the beginning of the study. The trial is registered on ClinicalTrials.gov NCT03755960. A report on the study protocol was already published earlier [14].

The randomized, controlled, two-armed, multicenter, parallel-group ACUDPN trial was conducted between February 2019 and April 2021 at the German Charité Universitätsmedizin Berlin and at an outpatient clinic for TCM at the University Medical Center Hamburg-Eppendorf in Hamburg, Germany. 

Patients were recruited through poster advertising on public transport and flyers in medical practices, podiatrists’ offices, and in strategic areas on the university campus and university hospitals. Patients received free acupuncture treatment.

Patients with type II diabetes of any sex, aged between 18 and 80, with a clinical diagnosis of DPN, were invited to participate. Other important inclusion criteria were overall complaints of at least 40 mm on a visual analog scale (VAS 0 mm = no complaints to 100 mm = worst imaginable complaints), no adjustments in medications related to DPN in the past 4 weeks, and pathological nerve conduction parameters regarding the suralis nerve (SNAP < 6 µV or nerve conduction velocity < 42 m/s); the absence of other causes for peripheral neuropathy. Participants were excluded if they had severe polyneuropathy with paresis of proximal muscles, obesity BMI > 35 kg/m^2^, ongoing anticoagulation, bleeding tendency due to thrombocytopenia, severe peripheral arterial occlusive disease, gangrene and ulcers in lower limbs, traumatic damage to nerves or vessels in the lower leg, used opioids or suffered from drug, alcohol, or medication abuse, or used ongoing nonpharmacological therapies for DPN such as psychotherapy or physical therapy. Patients were asked not to initiate other treatments for DPN-related symptoms during the study to avoid confounding. They had to be not pregnant or breastfeeding and had to provide written and verbal consent to participate in the trial.

During the first month of screening eligibility criteria, age and BMI were adjusted to increase the number of eligible patients from max age 75 to max age 80 and from max BMI 30 kg/m^2^ to max BMI35 kg/m^2^. Patients were randomly assigned at a 1:1 ratio in two groups (acupuncture or waiting list) using a computer-generated randomization list (prepared by SAS 9.4, SAS Institute Inc., Cary, NC, USA). The randomization list was kept at the study center in Berlin and was not accessible by the enrolling study physician. It revealed only one result at a time. If the study physician found a subject to be eligible for participation, a study nurse conducted the randomization and notified the study physician of the result by phone. Patients and physicians were not blinded regarding treatment allocation. Statisticians were blinded. 

Patients were enrolled in the study for a period of 24 weeks. The intervention group received a total of 12 acupuncture sessions in 8 weeks, with subsequent 16 weeks of follow-up. Details of the intervention have been published previously [14]. 

The control group was put on a waiting list for acupuncture treatment and completed a series of follow-ups for the first 16 weeks. Acupuncture treatment for the control group started at week 16 and ended at week 24 with the final follow-up. The same acupuncture treatment protocol (as in the intervention group) was applied. Routine care was continued in both groups throughout the trial. The study was designed to examine the overall effects of acupuncture on DPN. Therefore, we compared acupuncture in addition to routine care only. 

**Figure 1:**
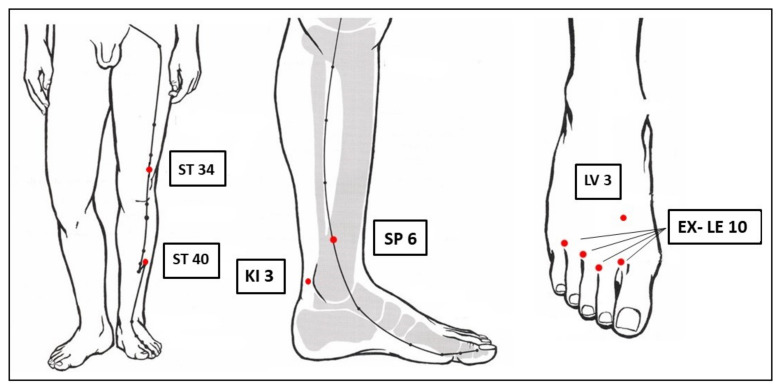
Mandatory acupuncture points in red. Figure modified with permission from Gabriel Stux, Textbook and Atlas, Springer [15].

The semi-standardized ACUDPN treatment protocol was developed based on Chinese medicine (CM) theory in consensus with German experts of CM. It included mandatory bilateral acupuncture of ST 34, ST 40, SP 6, KI 3, LV 3, and the four EX-LE-10 Bafeng (Figure 1). If necessary, GB 34, GB 39, GB 41, SP 4, SP 9, SP 10, KI 7, ST 36, and ST 41 could be added, as well as a heat source over the toes in case of very cold feet. All acupuncture points were on the lower extremities. A minimum of 18 needles was used per session and a maximum of 24.

The acupuncture treatment was carried out with sterile, single-use, stainless-steel 0.25 × 30 mm (manufactured by Dong Bang AcuPrime) and 0.25 × 40 mm needles (manufactured by PHOENIX). The skin was disinfected before needle insertion. Depending on the anatomical site and tissue, needles were inserted 1–2 cm perpendicular to the skin and rotated until the achievement of the needle sensation (De Qi). There was no manual stimulation after that. Needles were removed after 25 min. Acupuncturists were trained and certified with at least more than 120 h of experience.

All outcome parameters were assessed at weeks 8, 16, and 24 for both groups. Additionally, during the first 8 weeks of the study, both groups were asked to record their weekly VAS overall DPN-related complaints and VAS pain in their diaries.

The primary outcome parameter was the difference in the overall DPN-related complaints, measured with a 0–100 mm VAS (0 = no complaints, 100 = worst imaginable complaints) at 8 weeks between the groups. 

The secondary outcome parameters during the first 8 weeks were changes in VAS overall DPN-related complaints and VAS pain intensity, measured weekly from baseline to week 8. They were reassessed at weeks 16 and 24. The following parameters were assessed at weeks 8 and 16 between the groups: the Neuropathic Pain Symptom Inventory (NPSI), assessing subdimensions of neuropathic pain on an 11-point scale, changes in the German affective dimension of pain scale “Schmerz Empfindungsskala” (SES), the assessment of the general health-related quality of life using Short Form-12 (SF-12), as well as the disease-specific quality of life with the diabetic peripheral neuropathic pain impact (DPNPI) score, and the 7-point patient global impression of change (PGIC) scale. An intragroup analysis for the acupuncture group assessed the long-term effects until week 24. 

Patients were asked for adverse events in their diaries and at treatment appointments. Occurring adverse events were graded for severity and classified as treatment or nontreatment-related. Withdrawals and dropouts with reasons were documented. 

The sample size of 90 patients (45 per group) was calculated on the basis of an MCID of 1.5 points on a VAS and to provide 80% power. A total of 15% was added to account for dropouts, so 110 patients were planned. The changes in the overall number of DPN-related complaints (measured by the VAS) between the baseline and week 8 were used as the primary outcome parameter. The primary analysis of the primary endpoint was conducted with an analysis of the covariance (ANCOVA). The treatment group (acupuncture/control) and study center were included as the fixed-effect factors in the model, and the baseline value of the overall number of VAS-DPN-related complaints was used as a fixed covariate. The adjusted means were derived from this model and presented along with the two-sided 95% confidence intervals in each treatment group and the *p*-value of the group comparison (significance level of 5%, two-sided). The calculation was performed using the full analysis set (FAS) based on the intention-to-treat principle (ITT), evaluating each patient in the treatment group as randomized without the replacement of missing values. All further analyses were considered exploratory, without adjustment for multiple testing.

Similarly, the secondary outcomes were analyzed with an ANCOVA or logistic regression (depending on the scale of the outcome), including the treatment group and study center as fixed-effect factors and the respective baseline value (where applicable) as a fixed covariate. A subgroup analysis determined the effects of acupuncture on the subdimensions of neuropathic dysesthesias, such as tingling, numbness, and pain.

Data assessment was performed using SAS for Windows, Version 9.4 or higher (SAS Institute, Cary, NC, USA), SPSS version 26 or higher (IBM SPSS Statistics for Windows, Armonk, NY, USA: IBM Corp).

We conducted semi-structured qualitative interviews with 10 study participants to learn more about the subjective experience with DPN, medical care, acupuncture treatments, and study participation. The sample included participants from both groups who had completed the acupuncture treatment at least 1 week prior and were selected based on age, study group, and gender. Participants were recruited on a rolling basis as they completed the acupuncture treatment. Thus, the participants of the control group differed from the RCT because they were interviewed after receiving acupuncture and not during their waiting period. All those invited to the interview agreed to participate.

All interviews were recorded, transcribed, pseudonymized, and analyzed deductively and inductively using qualitative content analysis. The analysis was carried out with MAXQDA^®^ Standard 2018 (18.2.4) software. 

The results of the quantitative and qualitative evaluation were triangulated to possibly supplement and deepen the results. Detailed descriptions of the methods and results of the qualitative study and the triangulation will be published separately.

Due to strong restrictions on research with direct patient contact caused by the COVID-19 pandemic, the trial was terminated prematurely. Consequently, the previously calculated sample size of 110 patients (90 patients (45 per group) to provide 80% power plus 15% to account for estimated dropouts) was not reached. 

Originally, we planned to include “study center” as a fixed effect in the statistical models for the primary and secondary endpoints. However, due to the smaller sample size, the study center was not included as a fixed effect in the statistical models for primary and secondary endpoints in the predefined statistical analysis plan. Instead, the study center was included as a random effect in the analyses. The inclusion of a patient with an HbA1c below 6.5% represented a further protocol deviation with no impact on statistical analysis.

## 3. Results

**Figure 2:**
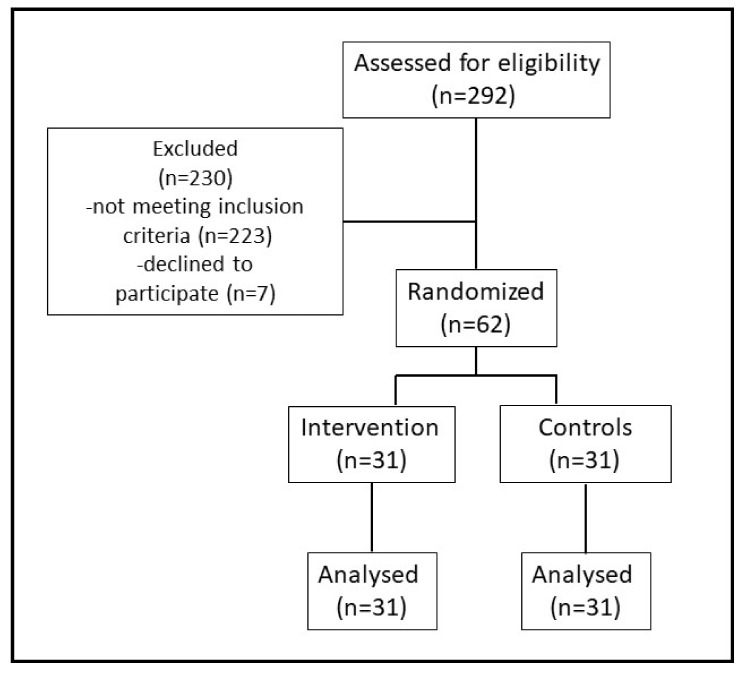
The ACUPN CONSORT study flowchart.

In total, 292 patients were screened for eligibility, and 230 did not meet the eligibility criteria. The main reasons for not being eligible for the trial were DPN-related causes other than type II diabetes, anticoagulation, age over 80, the use of opioids, a BMI higher than 35, and nerve conduction measurements that did not meet the inclusion criteria (see Figure 2).

The study did not recruit the planned number of patients due to its early discontinuation over the COVID-19 pandemic, in which patient contact was limited and reduced. A total of 62 patients met the inclusion criteria and were randomized into the intervention or control group (31 patients per group), with five participants having dropped out before the end of the study, three patients discontinued the trial in the intervention group, one due to increased tingling during the acupuncture, and two because of the COVID-19 pandemic during the follow-up; in the control group, two patients dropped out during follow-up because of the COVID-19 pandemic.

- Baseline parameters and demographics

At baseline, there were no relevant differences in the demographic characteristics, comorbidities, or concomitant medications between the two groups (Table 1). The higher number of men in the cohort reflects the epidemiology of the condition, which has a higher prevalence in men. Neither did the clinical inspection of the feet (regarding the condition of the skin: petechiae, ulcers, hypercornification, and palpability of pulses) reveal any differences between the groups (data not shown). The proportion of patients with a longer duration of neuropathy was higher in the waiting group (61.3%) compared with the acupuncture group (45.2%). Three patients in the acupuncture group and no patients in the control group had received acupuncture treatments against neuropathy in the past. 

The expectation regarding the effectiveness of acupuncture globally was not different between the groups, though more patients in the acupuncture group (80.6%) than in the control group (51.6%) expected a marked improvement in their DPN symptoms through the acupuncture treatment when asked at baseline. In the acupuncture group, 17 patients had DPN-specific medication at baseline vs. 22 in the control group.

- Primary and secondary outcomes

**Figure 3:**
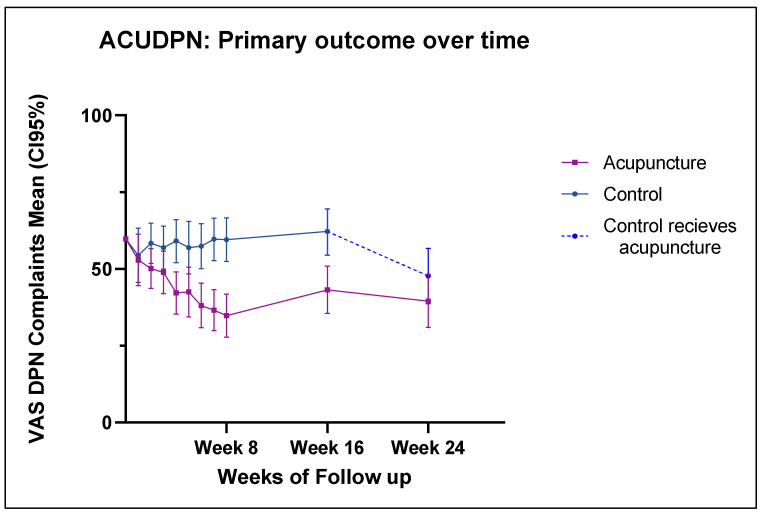
VAS overall related complaints values (mean and CI95%) from baseline to week 24. Controls received acupuncture intervention from week 16 to 24.

At the end of the intervention (week 8), the difference between the groups regarding VAS overall complaints was 24.7 mm (95% CI 14.8; 34.7, *p* < 0.001) in favor of the acupuncture group. The change in VAS overall complaints at week 8 compared to baseline was 34.8 (95% CI 27.8; 41.8) in the acupuncture group vs. 59.5 (95% CI 52.4; 66.6) in the control group (see Figure 3).

Subgroup analysis was conducted for the primary endpoint by the duration of neuropathy (>5 or ≤5) and BMI (>30 or ≤30). We observed no effect of the subgroups on the primary endpoint. The analysis for the remaining previously defined subgroups was not carried out due to an insufficient number of participants per group. 

Secondary outcomes

The effect for VAS overall complaints was still persistent at week 16, with a clinically meaningful difference of 18.9 mm (CI 95% 8.1; 29.8, *p* < 0.001) between the groups in favor of acupuncture. 

**Figure 4:**
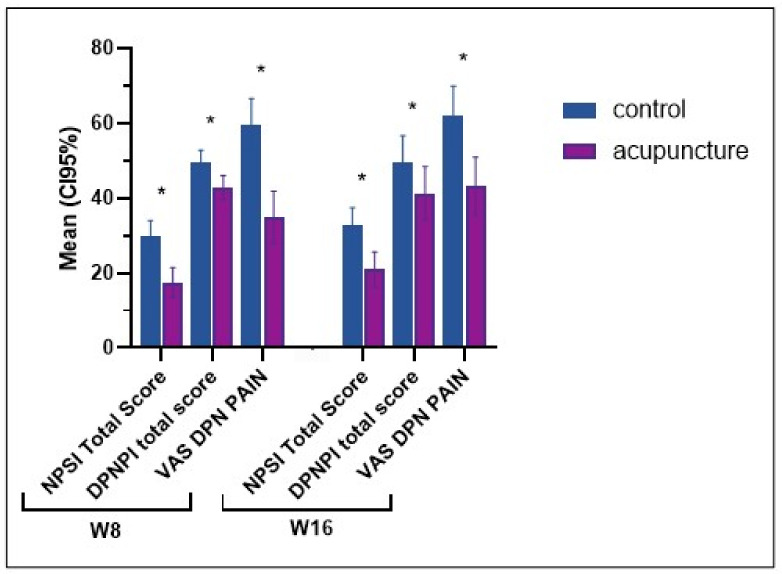
Secondary outcomes: NPSI: neuropathic pain symptom inventory, DPNPI: diabetic peripheral neuropathic pain impact measure, VAS: visual analog scale pain. High scores mean more DPN symptoms; the “*” indicate differences with a *p* < 0.05.

At week 8, the VAS pain showed a clinically meaningful difference of 28.7 mm (CI 95% 17.5; 39.9, *p* < 0.001) between the groups in favor of acupuncture. The effect observed in week 8 persisted into week 16, with a difference of 25.1 mm (CI 95% 13.8; 36.4, *p* < 0.001) between the groups. The NPSI, the SES, and the disease-specific impact on quality of life (DPNPI) scores also showed clinically meaningful differences between the groups in favor of the acupuncture in week 8 (*p* < 0.05); the effects persisted into week 16 (*p* < 0.05) (Table 2 and Figure 4). 

The SF-12 score did not reflect the results from the DPNPI and did not reveal clinically meaningful differences in favor of the acupuncture group, neither at week 8 nor at week 16 (Table 2).

The supplementary analysis of the weekly VAS data (from the patient diaries) showed that the first differences between the groups were found starting from week 4 onwards, with a difference of 16.7 mm (CI95% 4.8; 28.6, *p* < 0.05) in VAS overall complaints and of 16.9 mm (CI95% 7.1;26.8, *p* = 0.001) in VAS pain. 

From week 16 on, the control group received acupuncture, so an intergroup comparison for the final follow-up for week 24 was not possible. 

The reduction in VAS overall complaints and VAS pain in the control group (after receiving acupuncture from week 16 on) was 13.5 ± 19.6 and 13.5 ± 21.6, respectively, compared to week 24. On average, the reduction was slightly smaller than that observed in the treatment/acupuncture group from baseline to week 8, but it exceeded the MCID.

An explorative pre–post comparison for the acupuncture group showed that the reduction in the VAS overall complaints persisted, with a clinically relevant mean of 19.4 mm (baseline (SD) 58.5 ± 11.9 vs. 39.1 ± 23.8 at week 24. Additional pre–post comparisons of outcomes after the acupuncture treatments in both groups can be found in the Appendix A (Table A1).

The PGIC at week 8 showed that in the intervention group, 25 patients reported improvements, five reported no change, and one reported worsening, whereas in the control group, at week 24 (at the end of their acupuncture treatments), 21 participants reported improvements, five reported no change, and three reported the worsening of global symptoms. For further evaluations of the outcome parameters in the control group after acupuncture, see supplement. 

No meaningful changes were found in the amount of medication taken in either group; in the acupuncture group, 17.6% of patients reduced their medication during the 8 weeks of the study intervention and another 11% until week 16. In the control group, 13.6% of patients had reduced their on-demand medication at week 8 and 4.5% more at week 16. 

During 660 acupuncture treatments administered over the course of the study, 43 adverse events in total were related to acupuncture; of those, 18 were small hematomas at single needling sites, and seven were transient paraesthesia, with one of these leading to a patient dropping out of the intervention group. Additionally, five patients reported transient pain at needling sites, five patients reported tiredness after single treatments, four had a transitory intensifying of DPN-related symptoms that resolved in the days after a single session, one patient reported cramps at thigh muscle after needling, one felt light-headed, and one reported itching at the needling site. 

The details of the qualitative substudy and the triangulation will be published separately. Most interviewed patients reported an improvement in DPN-related symptoms, particularly numbness, pain, and increased mobility. This was reflected in our primary and secondary outcomes. Some interviewees reported that the effect was stronger when receiving two treatments per week rather than only one. Most of the participants favored further acupuncture treatments and were satisfied with our medical assistance, which they reported was easily integrated into their everyday lives.

- Interpretation of results

In this randomized controlled clinical trial, 12 acupuncture treatments over 8 weeks led to a significant reduction in overall DPN complaints. Furthermore, the patients achieved a clinically meaningful reduction in pain and reported a decrease in the neurological pain symptom inventory (NPSI) and the affective dimension of pain score (SES). Moreover, there was an improvement in the disease-specific impact on the quality of life, primarily due to an improvement in sleep and pain (DPNPI). The clinical effects persisted in the intervention group after the last acupuncture. Two months after the end of the intervention, overall DPN complaints were still reduced in a clinically meaningful manner compared to the control group. They were still detectable for up to four months after the end of the acupuncture in the intervention group. 

## 4. Discussion

Previous studies have used only pain or neurography as the primary outcome to study the effect of acupuncture on DPN-related symptoms [11,12]. But since DPN produces a variety of neuropathic symptoms (some of them painless), we chose a scale for overall complaints as the primary outcome instead. This allowed for a better estimation of the effect of acupuncture treatment. In addition, our study is characterized by a particularly long follow-up of 16 weeks, which is unmatched by previous trials. The lasting effects in overall complaints, pain, and symptom scores up to week 24 promise persistent symptom relief. In patient care, this effect could possibly be prolonged by repeated acupuncture treatments. However, this common practice has not been investigated yet.

This trial added to the evidence that, despite their chronic underlying condition, patients with neuropathy could benefit from acupuncture regarding their neuropathic symptoms. The study also provided a (so far) unique follow-up length and data on the intermediate and long-term effects of this specific acupuncture treatment regimen. Further, through the provision of acupuncture treatment in the waitlist control, the trial provided data on the reproducibility of the results. Finally, despite the study being underpowered due to its early discontinuation over the COVID-19 pandemic, the primary endpoint results still reached significance.

However, in the absence of a sham procedure, including the blinding of patients, certain effects should be considered. The placebo effect should be addressed at this point, which could have influenced the outcomes. The fact that patients knew they were getting the treatment may have influenced their evaluation of the VAS and all the other subjective scores in both groups. Further factors could have influenced the results; in the acupuncture group, more patients had experience with previous acupuncture treatments for various reasons (58.1% vs. 51.6%), and 9.1% vs. 0% had previous acupuncture against neuropathy; moreover, the acupuncture group showed higher efficacy expectations due to acupuncture at baseline. However, based on another publication, this high expectation might not have necessarily had a strong influence on the outcomes [16]. The comparable treatment effects of the control group from week 16–24 should be considered as well, which add important aspects of the replicability of effects.

Besides, the trial does not provide information on the specificity of the acupuncture points. A placebo-controlled study would be necessary to further evaluate the impact of acupuncture on this condition, and depending on the choice of the placebo (sham needle vs. sham points), the specific effects of the selected points might be called into question, as sham acupuncture with needling in the proximity of the real points might elicit comparable local effects.

The patients in the control group suffered longer and from more severe neuropathy at baseline than in the intervention group, which may explain why this group did not show the same extent of benefit after receiving the treatments from weeks 16 to 24. The difference was otherwise somewhat minimized, with a higher baseline complaint score in the intervention group. Further, it is not possible to say whether the reduction in DPN-specific medication in the intervention group persisted between weeks 9–15, as we did not collect such data for this period. 

The study was conducted exclusively on patients with type 2 diabetes; thus, further studies are required to assess the transferability of the effects to type 1 diabetes-induced polyneuropathy. 

The results are consistent with previous sham-controlled studies, showing an improvement in neuropathic symptoms and sleep quality [11,13]. The results of our nerve conduction assessments, along with clinical examination scores, will be presented and discussed in another publication. The results of the quantitative and qualitative analysis align, as most interviewees in the qualitative study reported positive effects from acupuncture on their DPN symptoms.

The effect on health-related quality of life measured by the SF-12 was not consistent with the otherwise positive results of the DPNPI in favor of the intervention group and with the findings of previous studies, which reported either improvements or no significant changes in the quality of life compared with the control group [11,12,16]. 

The control group experienced smaller effects right after the acupuncture treatment than the acupuncture group did. This could be related to the fact that the waiting list group had a higher proportion of severe neuropathy and a higher percentage of patients with neuropathic symptoms for more than 5 years. This may indicate that acupuncture should rather be recommended for moderate and mild symptoms and that patients should receive acupuncture as early as possible in the course of the disease. We ran a subgroup analysis to determine the effects, but due to too small sizes of the subgroups, the results of the analysis were not conclusive.

This study has shown that acupuncture is a safe and valuable addition to routine care. The study population represented a quite typical sample of patients with DPN. Over 50% of the patients had used acupuncture in the past for various reasons. Acupuncture has been popular in Germany for many years, and in major cities like Berlin and Hamburg, it is broadly available. In addition, statutory health insurance companies cover acupuncture treatment for chronic low back pain and chronic pain due to knee arthrosis, which is frequently used by patients. Our treatment protocol is a practical guideline for everyday clinical practice, as it provides a thought-out treatment concept while leaving room for individual therapy approaches. Our multicenter trial showed that our protocol could be applied in different treatment contexts. As a result of our wide eligibility criteria, our findings may be applicable to a broad population.

Future acupuncture trials for DPN should integrate objective parameters, such as vibration fork testing or nerve conduction studies. Further placebo-controlled studies could investigate the specific effect of acupuncture points on this condition or if a repetition of only a few acupuncture treatments could prolong the significant improvements over a longer period and, therefore, represent a useful addition to routine DPN care.

## 5. Conclusions

Acupuncture leads to significant and lasting reductions in DPN-related complaints compared to routine care and is well tolerated with minor side effects. More high-quality clinical trials are needed.

## Figures and Tables

**Table 1:** Baseline characteristics of trial participants.

Characteristics	Acupuncture Group (n = 31)	Waiting Group (n = 31)
Age mean (SD)	66.7 (7.6)	69.5 (7.2)
Age > 60 years	24 (77.4%)	27 (87.1%)
Male	25 (80.6%)	24 (77.4%)
Cardiovascular comorbidities	27 (87.1%)	27 (87.1%)
Specific DPN-related medication	17 (54.8%)	22 (70.9%)
Previous treatment Acupuncture against DPN Symptoms	3 (9.7%)	0 (0.0%)
Previous Acupuncture treatment for other reasons	18 (58.1%)	16 (51.6%)
Study therapy was estimated to be effective	27 (87.1%)	24 (77.4%)
BMI > 25 kg/m^2^	27 (87.1%)	27 (87.1%)
Duration of neuropathy symptoms > 5 years	14 (45.2%)	19 (61.3%)
VAS overall complaints Mean (SD) Median (Range)	58.5 ± 11.9 54 (42–84)	61.2 ± 11.5 60 (40–81)
VAS pain Mean (SD) Median (Range)	49.3 ± 22.5 52 (2–84)	39.3 ± 25.1 50 (0–74)
NPSI Total intensity score Mean (SD) Median (Range)	28.1 ± 15.8 30 (4–63)	28.8 ± 20.5 23 (0–72)
SES global affective pain experience Mean (SD) Median (Range)	25.0 ± 10.9 22 (14–54)	24.0 ± 9.0 22 (14–46)
SES global sensory pain experience Mean (SD) Median (Range)	16.9 ± 5.9 15 (10–36)	17.0 ± 6.5 16 (10–31)
DPNPI total score Mean (SD) Median (Range)	45.0 ± 14.0 45 (20–72)	51.5 ± 12.7 49 (26–73)
SF-12 physical component scale Mean (SD) Median (Range)	40.6 ± 9.7 41 (22–55)	37.6 ± 8.6 37 (21–53)
SF-12 mental component scale Mean (SD) Median (Range)	50.8 ± 10.9 55 (24–65)	48.4 ± 10.3 49 (21–65)

Data are presented as mean (SD), mean and range, or frequency (percentage). Abbreviations: *n* = number of participants, VAS = visual analog scale, NPSI = neuropathic pain syndrome inventory, DPNPI = diabetic peripheral neuropathic pain impact measure, SES = German scale for affective component of pain, SF-12 = Short Form-12.

**Table 2:** Primary and secondary outcomes in week 8 and week 16.

Outcome	Week	N	Acupuncture Group Adj. Mean [95% CI]	Control Group Adj. Mean [95% CI]	Difference Adj. Mean [95% CI]	*p*-Value
VAS DPN overall complaints (0–100 mm)	8	61	34.8 [27.8;41.8]	59.5 [52.4;66.6]	24.7 [14.8;34.7]	<0.001
	16	60	43.2 [35.5;50.9]	62.2 [54.5;69.9]	18.9 [8.1;29.8]	<0.001
VAS DPN pain (0–100 mm)	8	60	27.5 [19.7;35.3]	56.2 [48.4;64.0]	28.7 [17.5;39.9]	<0.001
	16	60	34.2 [26.3;42.1]	59.3 [51.4;67.2]	25.1 [13.8;36.4]	<0.001
NPSI total score	8	61	17.4 [13.5;21.4]	30.0 [26.0;34.0]	12.6 [7.1;18.0]	<0.001
(0–100)	16	61	20.9 [16.2;25.6]	32.6 [27.8;37.4]	11.7 [5.0;18.4]	<0.001
SES affective pain (14–56)	8	61	22.9 [19.5;26.2]	26.0 [22.5;29.6]	3.2 [0.1;6.3]	0.045
	16	60	22.9 [19.4;26.4]	27.2 [23.5;30.9]	4.2 [1.3;7.2]	0.005
SES sensory pain (10–40)	8	61	15.2 [12.6;17.8]	17.5 [14.8;20.2]	2.4 [0.4;4.3]	0.018
	16	60	15.5 [14.3;16.8]	18.5 [17.2;19.8]	2.9 [1.1;4.7]	0.002
DPNPI total score (18–90)	8	61	42.8 [39.6;46.0]	49.6 [46.3;52.8]	6.8 [2.2;11.4]	0.005
	16	61	41.3 [34.2;48.4]	49.3 [42.0;56.6]	8.0 [3.4;12.6]	<0.001
SF12 physical comp. scale (0–100)	8	56	40.3 [35.7;44.8]	37.5 [32.9;42.1]	−2.7 [−6.7;1.2]	0.171
	16	56	42.1 [39.7;44.5]	37.6 [35.3;39.9]	−4.5 [−7.8;−1.1]	0.010
SF12 mental comp. scale (0–100)	8	56	46.0 [42.8;49.1]	47.5 [44.6;50.4]	1.5 [−2.8;5.8]	0.478
	16	56	48.5 [45.3;51.8]	49.2 [46.0;52.5]	0.7 [−3.4;4.8]	0.731

VAS = visual analog scale, NPSI = neuropathic pain syndrome inventory, SES = German scale for affective component of pain, DPNPI = diabetic peripheral neuropathic pain impact measure, SF-12 = Short Form-12. Higher scores indicate a worse outcome: VAS, NPSI, SES, and DPNPI. Higher values indicate a better outcome: SF12.

## Data Availability

Data are available under reasonable request to the corresponding author.

## Appendix A

**Table A1:** Explorative analysis of pre-post values of the control group in comparison to the intervention group (acupuncture from week 1–week 8; controls from week 16–week 24).

	Intervention Group Acupuncture from Weeks 1–8			Controls Acupuncture from Weeks 16–24		
	Pre	Post	Difference	Pre	Post	Difference
VAS overall complaints	58.5 ± 11.9	34.3 ± 21.1	24.2 ± 23.2	61.8 ± 19.6	48.2 ± 24.4	13.5 ± 19.6
VAS Pain	***48.4*** ± 22.4	29.7 ± 21.8	18.7 ± 22.4	54.1 ± 27.9	40.6 ± 30.9	13.5 ± 21.6
NPSI	28.1 ± 15.8	17.6 ± 14.1	10.5 ± 13.5	31.6 ± 20.4	26.1 ± 20.1	5.5 ± 15.0
SES affective pain	25.0 ± 10.9	23.8 ±10.45	1.2 ± 6.1	27.2 ± 11.0	22.8 ± 8.9	4.4 ± 7.7
SES sensory pain	16.9 ± 5.9	15.6 ± 5.0	1.3 ± 4.1	18.1 ± 7.5	16.5 ± 5.3	1.4 ± 5.0
DPNPI total score	45.0 ± 14.0	40.3 ± 16.4	4.7 ± 9.9	54.2 ± 14.8	50.9 ± 14.6	3.3 ± 8.8
SF12 physical comp. scale	40.0 ± 9.5	42.1 ± 10.2	1.7 ± 7.9	37.4 ± 8.3	37.4 ± 9.4	0.6 ± 5.2
SF12 mental comp. scale	50.7 ± 11.3	45.9 ± 12.3	3.8 ± 7.8	48.0 ± 11.0	47.5 ± 12.5	0.7 ± 6.8

VAS = visual analog scale, NPSI = neuropathic pain syndrome inventory, SES = Schmerzemp findungsskala German scale for affective component of pain, DPNPI = diabetic peripheral neuropathic pain impact measure, SF-12 = Short Form-12-Quality of Life. Higher scores indicate a worse outcome: VAS, NPSI, SES, and DPNPI. Higher values indicate a better outcome: SF12.
